# Changing the preschool setting to promote healthy energy balance-related behaviours of preschoolers: a qualitative and quantitative process evaluation of the SuperFIT approach

**DOI:** 10.1186/s13012-021-01161-9

**Published:** 2021-12-04

**Authors:** Ilona van de Kolk, Sanne Gerards, Anke Verhees, Stef Kremers, Jessica Gubbels

**Affiliations:** Department of Health Promotion, School of Nutrition and Translational Research in Metabolism (NUTRIM), Maastricht University, PO Box 616, 6200 MD Maastricht, The Netherlands

**Keywords:** Process evaluation, childcare, preschool, context, implementation, maintenance, environment

## Abstract

**Background:**

The Early Care and Education (ECE) setting plays an important role in the promotion of a healthy lifestyle in young children. SuperFIT is a comprehensive, integrated intervention approach designed to promote healthy energy balance-related behaviours in preschoolers. Insight in the process of implementation and the context in which SuperFIT was implemented supports the understanding of how the intervention works in practice. This process evaluation examined factors that influenced the implementation and maintenance, as well as the (perceived) changes in the ECE setting.

**Methods:**

A mixed-methods study was conducted. SuperFIT was implemented at twelve preschools in the south of the Netherlands. The process evaluation was performed among preschool teachers, managers of the preschool organisation, and implementers. Semi-structured in-depth (group) interviews, quantitative process questionnaires, the Child-care Food and Activity Practices Questionnaire (CFAPQ) and the Environmental and Policy Assessment and Observation (EPAO) were used to evaluate the implementation and maintenance of SuperFIT and the changes in the preschool setting. The interviews were analysed using a theoretical framework based on the Implementation Framework of Fleuren and Damschröder’s Consolidated Framework for Implementation Research. Descriptive analyses were performed on the quantitative data.

**Results:**

Various intervention activities were implemented in the preschool setting. Although the intention to maintain SuperFIT was present, this was hindered by time constraints and lack of financial resources. Important factors that influenced implementation and maintenance were incongruence with current practice, limited perceived capabilities to integrate SuperFIT in daily practice, group composition at the preschools, and the perceived top-down implementation. Organizational vision and societal attention regarding healthy behaviour in general were perceived to be supportive for implementation and maintenance. Predominantly, favourable changes were seen in the nutrition- and physical activity-related practices of preschool teachers and other aspects of the social preschool environment such as the use of play materials. Limited changes were observed in the physical preschool environment.

**Conclusions:**

Several factors influenced the implementation and maintenance of SuperFIT in the preschool setting. Some factors evolved over time from hindering to facilitating, emphasising the importance of allowing sufficient time for intervention implementation. SuperFIT changed mainly the social preschool environment.

**Trial registration:**

Clinicaltrials.gov, NCT03021980, date registered: January 16, 2017, prospectively registered

**Supplementary Information:**

The online version contains supplementary material available at 10.1186/s13012-021-01161-9.

Contribution to the literature

- Insight in the factors influencing the implementation process of health-promoting interventions within the ECE setting is important for understanding the success of these interventions. Currently, the focus of process evaluations is on quantitative implementation concepts.
- The implementation of interventions is influenced by the context of the implementation setting. Allowing the intervention to be adapted to its context is necessary to support the integration of interventions into daily practice.
- To allow for changes within the ECE setting, intervention implementation should be considered a long-term effort.

## Background

The Early Care and Education (ECE) setting has been recognized as an important setting for promoting healthy energy balance-related behaviours (EBRBs) in young children [1, 2]. Firstly, supportive nutrition and physical activity (PA)-related practices of ECE staff may promote healthy EBRBs in children [3, 4]. Secondly, availability of (outdoor) play spaces and a variety of play materials may support children’s PA [5–8]. Thirdly, availability of healthy food products can support children’s healthy dietary intake [9]. Lastly, policies can support activities within the ECE setting to promote healthy EBRBs [10, 11]. Consequently, attending childcare has been related to both increased and decreased risks of childhood overweight and obesity [12–15].

In recent years, an increasing number of interventions in the ECE setting have been developed and evaluated (e.g., [16–19]). Review studies of such interventions show their potential in changing children’s behaviour, although the available evidence is often limited [2, 20–22]. Taking a comprehensive approach (i.e., aiming at both nutrition and PA, and by involving parents) has been recognised as being important for the effectiveness of these interventions [21–23].

SuperFIT (Systems of Underprivileged Preschoolers in their home and preschool EnviRonment: Family Intervention Trial) is a comprehensive, integrated intervention approach being applied in the Netherlands [24]. SuperFIT aims to improve children’s EBRBs through changes in the sociocultural, physical, and political environments in both the preschool and home settings. An effectiveness study of SuperFIT showed no significant differences between the intervention groups and control group on BMI z-score, overall PA, and dietary intake (Harms LSE, Gubbels JS, Van de Kolk I, Bessems KMHH, Vanbelle S, Hahnraths MTH, et al: The effects of SuperFIT, a comprehensive, integrated intervention approach, on pre-schoolers dietary intake, submitted) [25]. However, preschoolers who participated in the full intervention (preschool and family component) showed significant positive differences with the control group in PA on preschool days and in sweet beverage consumption (Harms LSE, Gubbels JS, Van de Kolk I, Bessems KMHH, Vanbelle S, Hahnraths MTH, et al: The effects of SuperFIT, a comprehensive, integrated intervention approach, on pre-schoolers dietary intake, submitted) [25].

Insight in the processes that influence implementation may clarify why interventions fail or succeed in changing behaviour [26]. Several frameworks explaining implementation processes are available [26–28]. The role of context has become more important in intervention implementation [29, 30]. More emphasis is being put on the unique characteristics of the implementation setting, for example preschools, and how the setting functions as a complex system [30, 31]. Intervention implementation is given an extra dimension: to what extent was the intervention able to interact with the context of the system and able to ‘saturate’ the context of this specific setting [30]? A combination of quantitative and qualitative research methods is needed to provide insight in both determinants for implementation including context [32]. High implementation quality is important, as a significant *decrease* in steps/weekday was shown for children in kindergartens with low-quality implementation (i.e., low dose delivered/received, or satisfaction) of a previous PA-promoting intervention at childcare, compared to no significant change in the control group [16]. Such negative intervention effects were not shown with medium- and high-quality implementation [16]. Few process evaluations of interventions in the ECE setting are currently available, and those that do exist tend to focus on reporting quantitative implementation concepts such as dose delivered and fidelity [16, 33–36]. However, some studies also described factors influencing the implementation process. Negative factors were, for example, lack of time, lack of support from staff, interference with daily schedules, and low parental engagement [33, 36]. Support from the intervention provider was identified as a supportive factor for implementation [35, 36]. Insight in the factors that influenced the implementation process is pivotal. This can increase our understanding of what the intervention implementation looked like in practice and may shed light on the mechanisms underlying the observed changes [37]. The current study presents the process evaluation of the SuperFIT approach, specifically within the preschool (ECE) setting. Several research questions will be addressed: 1) How was SuperFIT implemented and maintained in the preschool setting and how was this received by actors within this setting?; 2) Which factors influenced implementation and maintenance?; and 3) What were the (perceived) changes in the preschool setting?

## Methods

### Research design

A mixed-methods design was adopted, using both qualitative and quantitative research methods. This process evaluation is part of a larger evaluation study, described in detail elsewhere [24] and prospectively registered (Clinicaltrials.gov, NCT03021980). The current study focussed specifically on the implementation and maintenance processes within the preschool setting. A process evaluation of these processes in the home setting is presented elsewhere [38].

### The SuperFIT approach

SuperFIT is a comprehensive, integrated intervention approach and a detailed description of its development and evaluation has been previously published [24]. Socio-ecological models and systems theories on behaviour were used as theoretical background [39–41]. SuperFIT was developed in cooperation with a local PA-providing organisation, a preschool organisation, and health promotion experts in a continuous process of co-creation and adaptation. The SuperFIT approach consisted of three components: a preschool-based component, a family-based component, and a community-based component.

Intervention strategies in the preschool-based component aimed to change the sociocultural, physical, and political environments [42]. Strategies to change the sociocultural environment focussed on PA and nutrition-related practices of the preschool teachers. Several training sessions addressing PA, nutrition and positive child rearing were organised. Each off-the-job training session was accompanied by coaching on-the-job to assist implementation in the workplace. The training sessions and coaching on-the-job were delivered by trained health brokers from the local PA-providing organisation. PA and nutrition cards were developed, containing easy-to-perform PA- or nutrition-related activities. To change the physical environment, a box with materials supporting active play (e.g., hoops, balls, trampoline) and nutrition-related materials (e.g., water tap and nutrition-related story books) was provided. The materials were matched to the PA and nutrition cards, ensuring that teachers would have the materials needed to perform the activities on the cards. In addition, PA- and nutrition-related materials matching specific needs of each preschool were provided. A complementary fruit and vegetables (F&V) delivery aimed to increase F&V variety (e.g., cherries, raspberries, beetroot, radish). In order to change the political environment, strategies aimed to update the nutrition policy (provision of water instead of sugar-sweetened drinks and healthy treats) and develop a PA policy (including recommendations on time spent on active and safe play) were initiated. SuperFIT activities started in April 2017 with the first off-the-job training, on-the-job coaching, and provision of PA- and nutrition-related materials. In May 2017, the additional F&V delivery started. In the fall of 2017, two more training sessions and coaching on-the-job were provided. SuperFIT activities ended in May 2018, with the conclusion of the F&V delivery.

SuperFIT also included a family-based component (a combination of family sessions and caregiver-only sessions) and a community-based component (development and distribution of a social map of PA possibilities) component, but these are not the focus of the current paper.

### Study setting and participants

SuperFIT was implemented at a convenience sample of twelve preschools in low socioeconomic communities from one preschool organisation in the south of the Netherlands [43]. In the Netherlands, preschool consists of half-day, formal childcare in which children aged 2-4 years are prepared for primary school in a playful manner [44]. The process evaluation was performed among preschool teachers, management of the preschool organisation, and implementers (health brokers from the PA-providing organisation). All participants provided (verbal) informed consent before participating in the study. The Maastricht University Medical Centre, Medical Ethics Committee reviewed and approved the evaluation study of SuperFIT (METC163022/NL58061.068.16).

### Data collection

**Fig. 1:**
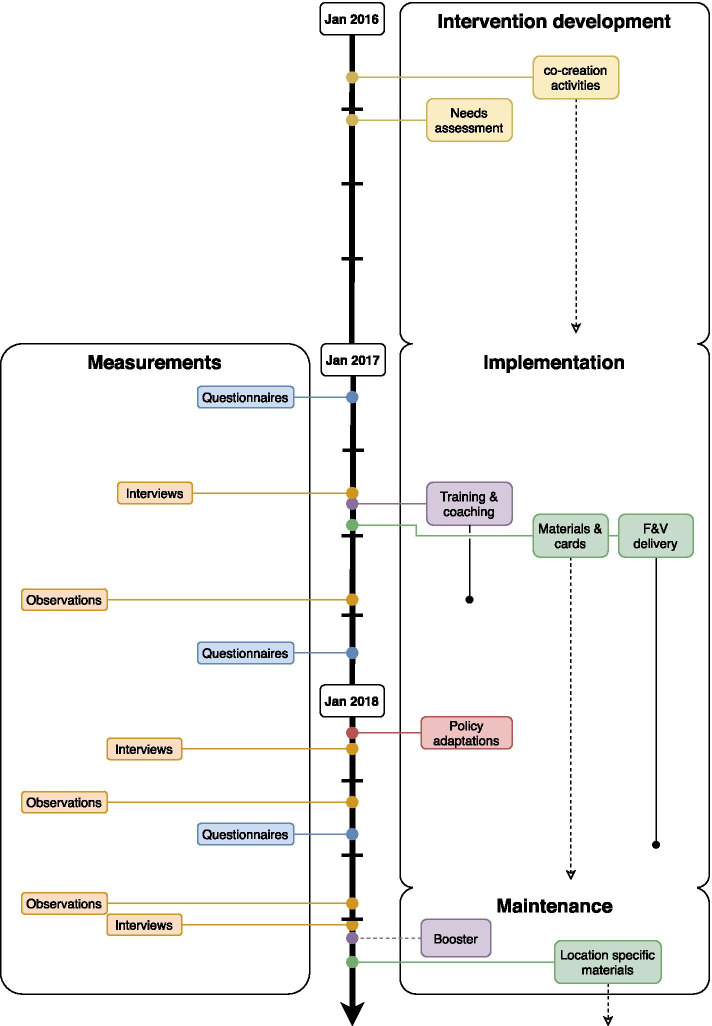
Timeline of the implementation of the preschool component of SuperFIT and the measurements of the process evaluation. Yellow= activities for the development of SuperFIT, purple= intervention activities aimed at the sociocultural environment, green= intervention activities aimed at the physical environment, red= intervention activities aimed at the political environment, blue= quantitative process measurements, orange= qualitative process measurements.  Indicates duration of an activity.  Indicates continuous activities or availability.

Data collection consisted of quantitative and qualitative measurements taken between January 2017 and November 2018 on several occasions during implementation and maintenance (see Fig. 1 for the timeline of implementation and evaluation). Following the RE-AIM framework [45, 46], implementation was operationalised as delivery of SuperFIT as intended. Factors influencing this process were studied. Maintenance was operationalised as the extent to which SuperFIT became institutionalised, i.e., part of the routine practices within the preschools after ending of the implementation activities [45, 46].

#### Interviews

Semi-structured in-depth group and individual interviews were performed by two researchers. During the initial phase of implementation (June/July 2017), interviews were conducted with preschool teachers and implementers. These interviews focused on development and implementation and served as input for the intervention activities that were still to come. At the end of the implementation phase (February/March 2018), in-depth interviews with preschool teachers were conducted to evaluate the implementation process. After the implementation phase (October/November 2018), interviews were conducted with preschool teachers, managers of the preschool organisation (both general and unit management), and implementers to evaluate the maintenance of SuperFIT. For each round of interviews, topic lists were developed. Topics included, for example, strengths and weaknesses of SuperFIT, the perceived role of the interviewee in nutrition and PA in preschools, and perceived changes (full topic lists are provided as Supplementary materials). All interviews were held in Dutch and audio-recorded.

#### Questionnaires

The Child-care Food and Activity Practices Questionnaire (CFAPQ) was used to measure preschool teachers’ nutrition- and PA-related practices [47]. The CFAPQ was filled in prior to, during and after implementation. The CFAPQ was adjusted to fit the Dutch preschool setting. Some items were omitted because they were not applicable for the preschool setting. In addition, for some items, examples provided were adjusted to better fit the preschool setting. An item to measure a PA-related practice is, for example, *‘How often do you play a sport or active game together with the children?’*. An item to measure the nutrition-related practices is, for example, *‘I model healthy eating for the children by eating healthy foods myself’*. All items were measured on a 5-point Likert scale, ranging from never to always or totally disagree to totally agree. Scale reliability was tested using Cronbach’s alpha (>0.50 was considered sufficient [48]). Items were deleted from the scales to achieve sufficient reliability. Final unreliable scales and deleted items were analysed as single items.

Preschool teachers were asked to fill in a quantitative questionnaire regarding the implementation (November/December 2017) and maintenance (May/June 2018) of SuperFIT. The questionnaire regarding implementation was based on the Client Satisfaction Questionnaire [49] and included a question such as *‘Did you find SuperFIT instructive?’*. In addition, specific questions on each intervention activity were asked. Factors influencing maintenance were measured using the Measurement Instrument for Determinants of Innovations (MIDI), adapted to fit the SuperFIT context [50].

#### Observations

Observations at the preschools were performed using an adjusted version of the Environmental and Policy Assessment and Observation instrument (EPAO) to assess the social and physical preschool environment [51]. The parts applicable to the Dutch preschool setting were incorporated (e.g., pre-break indoor play, break, and post-break outdoor play). Observations were performed on a group level and focused on preschool teachers’ behaviour. Observers were guided by questions such as ‘*Do the preschool staff take part in outdoor play activities?*’. In addition, questions related to the implementation were incorporated to assess the integration of SuperFIT within the daily activities of the preschool staff. For example, ‘*Were fruit and/or vegetables from the delivery divided between all children?*’. Observations were performed by the same researcher twice during implementation and once after implementation in nine randomly selected participating preschools, of which one preschool had two groups that were both observed. The sample of observed preschools remained consistent over the three measurements. Items reflecting non-supportive preschool staff behaviour were recoded and sum scores reflecting a supportive social environment for various activities (e.g., outdoor play, indoor play and snack time) were calculated.

In order to assess the physical preschool environment, separate observations were performed by a trained researcher using the EPAO [51]. Observations assessed, for example, indoor and outdoor play space, availability of fixed and portable play materials (indoors and outdoors), and the presence of screens. The observations of the physical environment were performed once prior to implementation, once during, and once after implementation. Where appropriate, sum scores were calculated to aggregate different questions into a summary variable, for example, total score of available portable play materials.

### Analyses

#### Qualitative data

**Fig. 2:**
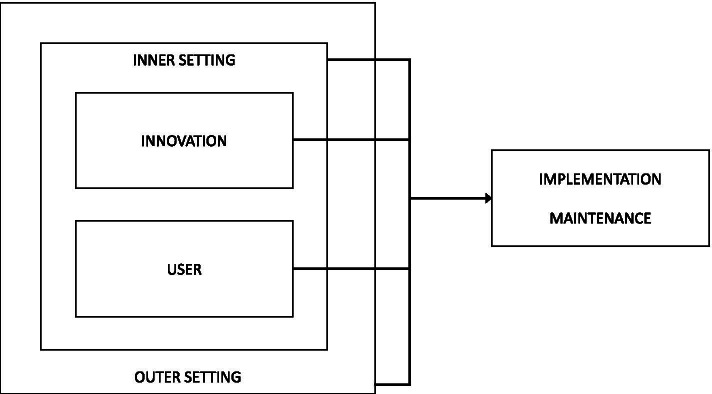
Theoretical framework for the process evaluation within the preschool setting; adapted from the Implementation Framework [28] and the Consolidated Framework For Implementation Research [27]

All interviews were transcribed verbatim and anonymised. A second researcher was consulted when words or phrases were unclear. To develop the coding tree, two researchers independently analysed 19% (6 of 32) of the interviews and held several consensus meetings. The Implementation Framework of Fleuren et al. [28] formed the basis for the coding tree. This framework was supplemented with the Consolidated Framework For Implementation Research (CFIR) to better reflect contextual factors influencing implementation and maintenance [27]. The main categories ‘characteristics of the intervention’ and ‘characteristics of the user’ from Fleuren et al. [28] were combined with the context-related factors in ‘the inner and outer setting’ from Damschröder et al. [27] (Fig. 2). The innovation was regarded as the preschool component of SuperFIT, the user was the preschool teacher. The inner setting involved direct contextual factors, for example, characteristics of the preschool organisation, the preschool itself, and the children attending the preschool. The outer setting included the broader context, such as societal influences. Following an abductive analysis strategy, researchers remained open for new insights in the data and analysis was not limited to determinants depicted in the framework [52]. Codes used were, for example, “complexity” or “relevance” (characteristics of the innovation), “self-efficacy” or “perceived advantages” (characteristics of the user), “formal reinforcement” or “time” (inner setting), and “society” or “cooperation with external parties” (outer setting). In addition, “implementation” and “maintenance” codes were used to code descriptions of what actually happened. Maintenance started after completing the last SuperFIT activity (F&V delivery, May 2018). One of the two researchers then analysed the remainder of the interviews. A final meeting between the researchers was held to discuss any difficulties that arose during analysis. NVivo 12.0 (QSR International, Doncaster, Victoria, Australia) was used to support data analysis.

#### Quantitative data

The quantitative data (from questionnaires and observations) were entered and cleaned before analysis. The scores on the CFAPQ of preschool teachers were aggregated to achieve a preschool-level score (N=12). Descriptive analyses were performed per measurement time point, on the data of the CFAPQ and observations. The sample size did not allow for further statistical testing and mean differences are presented. Analyses were performed using SPSS Version 25.0 (IBM, Armonk, NY, USA).

## Results

### Participants

A total of 32 interviews were held with a total of 49 participants, as some of the interviews included multiple participants. The interviews lasted 42 minutes on average (see Table 1 for participants’ characteristics). Thirty-one preschool teachers (response rate (RR)= 96.9%) filled in the CFAPQ at the first measurement, 24 (RR= 75.0%) at the second measurement, and 25 (RR= 78.1%) at the third measurement. Data was available for at least one teacher of each preschool at all measurement moments. At the first measurement, 26 preschool teachers (RR=81.3%) filled in the process questionnaire, at the final measurement 25 preschool teachers (RR=78.1%) filled in the process questionnaire. Observations for the physical environment were done at all preschools on all measurement moments. For the social environment, observations were performed for 10 groups (76.9%).

**Table 1:** Participant characteristics for each measurement

Participant characteristic	Interviews (***N***=49)^**a**^	CFAPQ^**b**^ (***N***=31)	Process questionnaire (***N***=26)
Age in years (mean)	45.1	46.8	45.7
Female gender (%)	97.0	100.0	100.0
>10 years work experience (%)	55.6	61.3	59.2

^a^Characteristics of preschool teachers based on baseline quantitative questionnaires; two participants did not provide a baseline questionnaire. Other interviewees provided characteristics during the interview. ^b^*CFAPQ* Child-care Food and Activity Practices Questionnaire

### Implementation of SuperFIT

Various intervention activities were implemented during the implementation phase (April 2017 – May 2018). On average, 89.0% of the preschool staff attended one or more of the off-the-job training sessions. In total, 42 different types of F&V were delivered, all preschools received 20 different types of PA-related materials and ten types of nutrition-related materials.

#### Integration of SuperFIT in daily practice

It took some time for preschool staff to start implementing activities and/or using materials, and differences were observed between preschools (see also Supplementary Table S1). Nonetheless, a majority of the preschool teachers eventually used the materials. During the observations, on average around one SuperFIT PA-related material was used by staff and one to two PA-related materials were used by the preschoolers. If staff used SuperFIT nutrition-related materials (7 or 8 locations), they always used the water tap. Preschoolers used on average around one nutrition-related material, which was most often the nutrition-related toys. Over time, more SuperFIT materials became visible in the preschools, and more staff used PA-related cards. Nutrition-related cards were hardly used. Preschool staff also started demonstrating other initiatives to integrate SuperFIT into their daily practice. For example, they started thinking about how to further reduce sedentariness. Preschool staff also indicated that over time they became more flexible in their daily structures to allow for more (short) active games.

### Maintenance of SuperFIT

Maintenance was explicitly addressed from the start of the project. Both the PA-providing organisation and the preschool organisation intended to continue SuperFIT after the initial implementation phase. All preschool teachers indicated that SuperFIT had become a way of working for them and continued using the materials and some of the cards. They continued to reflect on their routines and structures in order to increase PA (e.g., removing chairs to decrease the amount and frequency of sitting) or support healthy nutrition (e.g., preparing food together with the children). The ending of the F&V delivery greatly decreased the variety of F&V offered, as preschools were again dependent on the F&V that children brought with them from home. However, serving only water had become a habit.

#### Dissemination of SuperFIT

For the preschool organisation, the maintenance phase also focussed on the dissemination of SuperFIT to its remaining ECE locations. The organisation formulated a vision regarding healthy behaviours in ECE and integrated this into their policies. Although two managers were trained as so-called ‘healthy childcare-coach’ (part of a nationwide initiative ‘Healthy Childcare’ in the Netherlands [53]), dissemination of SuperFIT was not centrally coordinated by the organisation. Therefore, *how* SuperFIT was disseminated depended heavily on the motivation and efforts of individual managers. Dissemination was further influenced by available resources (time and finances).

### The innovation (preschool component)

The preschool staff mentioned that the enthusiasm of the implementers sparked their own enthusiasm for SuperFIT during the implementation phase. The quantitative process evaluation (Supplementary Table S2) showed that preschool teachers on average thought that SuperFIT was a good programme, and they found it interesting and instructive. The implementers were perceived as qualified.

#### Relevance of SuperFIT activities

The preschool staff considered the SuperFIT training and coaching as the most relevant parts of the approach (see Table 2 for all facilitating and hindering factors). The training off-the-job was appreciated (7.0, scale 1-10) because it provided the opportunity for staff to share experiences. The coaching on-the-job was found particularly relevant (7.4, scale 1-10), because it helped in applying SuperFIT into practice. Further, preschool staff found it very important that the implementer experienced their daily struggles. Some managers and implementers stated that the F&V delivery and materials were the easiest to implement, as they did not necessarily require active behavioural change on the part of the preschool staff.

**Table 2:** Facilitating and hindering factors for the implementation and maintenance of SuperFIT in the preschool setting

Domain	Facilitating factor	Example quotes	Hindering factor	Example quotes
**Implementation**				
**Characteristics of the innovation: SuperFIT**	- Training off-the-job, in particular to share experiences with other teachers and experts. - Coaching on-the-job.	*‘It is nice that you can share experiences like “how do you handle that?” … and it was nice that [the coach] came here to observe and give tips.’ (PT)*	- Incongruence with current practice.	*‘It is nice to hear, but then if you cannot apply it at your own preschool, I think that’s a shame.’ (PT)*
	- Enthusiasm of the implementers.	*‘I liked it a lot, that you can use the expertise of others who are enthusiastic about their profession and you also get a piece of the pie.’ (PT)*	- No changes in the outdoor play area (e.g., provision of fixed outdoor play materials).	*‘I had hoped for more things for outside, the design … Our playgrounds are so boring.’ (PT)*
	- Supportive materials to implement changes within the preschool location (e.g., the water tap).	*‘We started with the tap. Let them tap water themselves … and not one child has asked for lemonade since.’ (PT)*	- Limited appropriateness for preschoolers (e.g., cards too difficult to understand, too exotic F&V).	*‘But passion fruit, with the seeds and all, and the papaya was also hardly eaten and then I think … this may be too much’. (PT)*
	- Ready-made activities (e.g., delivery of materials/F&V).	*‘If you do not have to put in that much effort, like the F&V delivery, then it is easy to implement’. (M)*	- Predominantly top-down development of the intervention.	*‘I thought it would have been good if a small group of preschool teachers discussed things together, some cooperation or consultation’. (M)*
	- Long overall duration of the programme.	*‘It has to sink in, you have to experiment a bit and then you see that you changed your approach into something that works … this does not happen after the first training’. (PT)*	- Lack of innovativeness (content of SuperFIT) for the preschool staff.	*‘I was not really inspired. I felt like much of the ideas that arose, that we already do that. It was not new to me.’ (PT)*
**Characteristics of the user: preschool teacher**	- Preschool staff’s previous experience with PA, through training or work experience.	*‘You have to be creative with it, that’s something you learn with experience.’ (PT)*	- Preschool staff’s initial negative attitude towards the programme and lack of motivation to participate.	*‘I also thought that everyone was a bit hesitant, like, let’s wait and see what’s coming at us, does this suit us?’ (PT)*
	- Awareness among staff about the purpose of the intervention.	*‘Eventually, we learned that it’s the little things that matter and then it is possible to integrate it.’ (PT)*	- Low outcome expectations of the preschool staff to change children’s EBRBs due to expected lack of cooperation of the children.	*‘I mean, we try, but they won’t even taste it and then I think: “what did we achieve?”’ (PT)*
			- Low self-efficacy of staff due to other tasks, and characteristics of their location or group.	*‘At the beginning I thought “Do we get something extra to do again? We already have so little time … ”’ (PT)*
**Characteristics of the inner setting: preschool organisation**	- Health promotion (PA and healthy nutrition) was part of the organisational vision.	*‘We were already thinking of promoting a healthy lifestyle within our organisation before SuperFIT, but we hadn’t started yet, so it [SuperFIT] was right up our alley.’ (M)*	- Characteristics of the preschool location (e.g., limited space, crowded arrangement of the room, and limited availability of an outdoor play area).	*‘[The room] is already full, it is too small.’ (PT)*
	- Support for SuperFIT within the organisation.	*‘It was always nice that some managers or even the director were present [at the training sessions for teachers] to see what was going on.’ (PT)*	- Limited time available with the children (i.e., limited opening hours).	*‘I need more time if I want to do everything and if I want to add this [SuperFIT] too, then I just need more time at the preschool.’ (PT)*
			- Group composition and characteristics of the children (e.g., language difficulties or age differences).	*‘It really depends on the group. Sometimes you can do everything and sometimes you can do nothing, that’s typical for preschoolers, right?’ (M)*
			- Lack of information provision from the organisation.	*‘I had hoped that [the organization] would have put more effort in making the preschool teachers enthusiastic about SuperFIT at the start … Preschool teachers did not know what SuperFIT was about.’ (I)*
**Characteristics of the outer setting**	- Preschool situated within primary school building that also supported health promotion.	*‘Because we’re part of a primary school that’s a “healthy school”, we’re also kind of obliged [to serve only water].’ (PT)*	- Limited access to the gym, due to requirements of the primary school.	*‘Look, it’s a cooperation with the primary school, we used to use the gym more often … now it has become a larger school, so we can do less.’(PT)*
	- Current attention for healthy nutrition and physical activity in society.	*‘A healthy lifestyle is increasingly being put on the societal agenda, it’s considered increasingly important, also due to the increasing prevalence of overweight.’(I)*	- Low perceived support from parents for healthy nutrition and PA within the preschool setting.	*‘It is a pity that you receive little feedback from parents, not only on this topic but also more in general.’(PT)*
			- Strict rules and regulations of the Community Health Authority, e.g., regarding safety or hygiene, limiting PA.	*‘Just this morning I was playing on the floor, but the floor isn’t that clean. And I started to think “What would the Community Health Service think about this?”’ (PT)*
**Maintenance**				
**Characteristics of the innovation: SuperFIT**	- Congruence with current practice.	*‘Then we realised that you could also look at it differently and indeed can integrate it in your normal routines, by doing things just a bit different. Then it is possible to integrate it.’ (PT)*	- Predominantly perceived top-down approach.	*‘The idea of SuperFIT was still conceived behind a desk … it’s not the result of a need from the staff. They didn’t state “we think our community needs to be healthier”. (M)*
	- Relevance for the preschools.	*‘I think that the things the preschool teachers are taught about how to be physically active with the children, are concrete things they can use.’ (M)*		
**Characteristics of the user: preschool teacher**	- Positive attitude towards creating a healthy environment for preschoolers.	*‘Then I think, at least they got that bit here … because we have a varied offer [of F&V], they can taste different things, that’s what we can give them.’ (PT)*	- Low outcome expectations of only changing the preschool setting, particularly the home setting should also be addressed.	*‘To what extent can you influence it? If it doesn’t address the home situation, then things won’t change.’ (PT)*
	- The promotion of healthy nutrition and PA was regarded as part of the job.	*‘As I see it, now we’re just doing it, because it’s become part of our daily schedule, it’s become routine.’ (PT)*	- Some beliefs about not wanting to ‘go over the top’ with promoting healthy behaviour, in particular related to healthy nutrition (e.g., healthy treats or other celebrations).	*‘I don’t believe it’s good to withhold everything from the children … a child’s birthday is a celebration and you should be allowed to give some sweets or a small biscuit. I think that should stay.’ (PT)*
	- High motivation to work on healthy nutrition and PA for preschoolers.	*‘There are other locations that can’t wait to start working with it, who say “give me those cards and materials and I’ll start with it!”’ (M)*		
	- Self-efficacy to be able to integrate SuperFIT activities into daily practice.	*‘At the beginning we thought: “How are we going to do this?” but eventually, yes, you have to look at it differently and then you are able to do it.’ (PT)*		
**Characteristics of the inner setting: preschool organisation**	- Organisational vision on healthy nutrition and PA.	*‘We have made it [healthy lifestyle] one of the spearheads of our organisation.’ (M)*	- High workload and competition between the different tasks across the organisation.	*‘I think we can do much more with it [SuperFIT] if you get the time and space to work on it... now you have to do it in-between other things and then you can’t implement it properly.’ (M)*
	- Formulation/reformulation of organizational policies related to nutrition and PA.	*‘What we formulated in our vision three years ago is now in black and white in our policy: that we want to promote a healthy lifestyle.’ (M)*	- Availability of resources such as time and money.	*‘Do we have money for it? That discussion is being held now.’ (M)*
	- Training of managers and ‘Healthy Childcare Coach’.	*‘We talk about “healthy childcare” now, there is a plan of action regarding “healthy childcare”, and there was some training regarding “healthy childcare”’. (M)*	- Group composition and characteristics of the children (e.g., language difficulties or age differences within the group).	*‘It may influence the integration of SuperFIT: if everyone speaks the same language it’s a lot easier, compared to a group of 16 children with 10 different nationalities.’ (M)*
**Characteristics of the outer setting**	- Current societal views on healthy nutrition and PA.	*‘If you look at society, then everything is healthy this and healthy that.’(PT)*	- Low perceived support from parents for healthy nutrition and PA within the preschool setting.	*‘Parents do influence [implementation], if you want to do a lot and parents keep saying: “we don’t want that”, that has its influence.’ (M)*
	- Much attention towards healthy nutrition and PA within ECE setting.	*‘You just have to open a trade journal and it’s all about physical activity and healthy lifestyle, in that way it’s in tune with the spirit of the times.’ (M)*	- Lack of cooperation between the primary school and the preschool in health-promoting initiatives.	*‘Well, the primary school is renovating the playground, but they did not consult us … so I don’t know what it’s going to look like. It’s a shame they didn’t cooperate with [preschool organisation].’ (PT)*
	- Alignment of policies and activities between preschool and primary school.	*‘We are often located within primary school buildings and you follow the policy of the primary school. So, if birthday treats are not allowed at primary school it is easier to implement such a policy in preschool.’ (M)*		

*I* Implementer, *M* Manager, *PT* Preschool Teacher

#### Incongruence with current practice

During the implementation phase, preschool staff experienced incongruence between SuperFIT and their current practice. Due to time constraints, staff also felt that they were being forced to make choices between activities. However, transferring into the maintenance phase, staff increasingly recognized that SuperFIT was an add-in programme rather than an add-on one, facilitating integration within practice. Perception of barriers that were important during the implementation phase (e.g., limitations in the physical environment) decreased, and this assisted staff in integrating SuperFIT into their daily practice. Staff indicated that time was needed to integrate SuperFIT into their daily practice. The duration of the programme allowed for this, although time constraints remained important in the maintenance phase.

During the implementation phase, some SuperFIT activities were perceived to be inappropriate for the preschoolers, such as some of the cards (too difficult) and the F&V delivery (too ‘exotic’). However, in the maintenance phase, SuperFIT was described as being relevant for the preschoolers as it helped them get acquainted with new tastes and promoted PA (Supplementary Table S2).

#### Lack of innovativeness

Not all SuperFIT content was experienced as innovative or relevant, and often preschool teachers felt that it was not *them* that needed to change. Some aspects were lacking in SuperFIT, such as in-depth discussion of topics during the training (e.g., nutritional value of food) or changes to the outdoor play area.

#### Bottom-up or top-down

It was suggested that a stronger bottom-up approach, i.e., involving preschool staff more from the start of the development, might have resulted in a better fit of the different preschool activities. This factor was mentioned for both the implementation and maintenance phase. An anticipated barrier, predominantly by managers and implementers, was preschool staff not being willing to participate in such bottom-up processes.

### The user: preschool staff

The majority of the preschool staff were sceptical at the start of the implementation phase and lacked motivation to participate. However, an increased awareness among preschool staff about the goals and purpose of SuperFIT changed their attitudes. They became more enthusiastic and willing to integrate SuperFIT into their daily practice.

#### Attitude and motivation

In the maintenance phase, most participants expressed that they felt it part of their job to promote healthy nutrition and PA, and were convinced that they could make a difference for the children. The idea was expressed that the ECE setting served as an example for parents, and that it was a place where children could at least become acquainted with healthy nutrition and PA. However, the influence of the home environment was also recognised as a hindering factor for changing preschoolers behaviour. All participants remained motivated to continue with SuperFIT in their work, although not all barriers were resolved (e.g., limited time and resources) and it was not felt necessary to change all things (e.g., birthday or Christmas celebrations).

#### Outcome expectations

Preschool staff were surprised how easily the preschoolers switched to water, but they found it hard to get them to taste the new F&V. This was further hampered by staff’s low outcome expectation, as they did not expect it to help preschoolers eat more F&V since the produce did not fit preschoolers’ preferences. On the other hand, the F&V delivery was appreciated the most of all SuperFIT activities by the preschool staff (average appreciation 8.4, scale 0-10) and was reckoned the most successful aspect of SuperFIT.

#### Self-efficacy

Many of the preschool teachers stated that they did not always feel capable of implementing SuperFIT. Their reasons were predominantly related to their other tasks, characteristics of their location (e.g., limited space to use play materials), and fear of children hurting themselves or others. In the maintenance phase, increased self-efficacy supported the integration of SuperFIT into the daily practice of preschool staff in the longer run. This was also reflected in the quantitative process evaluation (Supplementary Table S1). They increasingly felt that it was something they were able to incorporate in their daily practice.

### The inner setting

SuperFIT fitted well with the vision of the organisation on healthy nutrition and PA in the preschool setting. Therefore, preschool staff felt that the organisation was committed to SuperFIT, which was reflected in the presence of management at the different activities. In the maintenance phase, this vision supported the formulation/reformulation of policies and the initiation of several organisational processes to maintain SuperFIT, such as the training of two managers to become ‘Healthy Childcare Coach’.

Managers had a preference for PA-related activities rather than nutrition-related activities. Nutrition was regarded more difficult to change, and healthy nutrition was considered to be an ambiguous subject.

#### Characteristics of the preschool group

Several characteristics of the preschool groups that influenced the implementation and maintenance phases were mentioned. Age differences between the children, language issues, (motor) developmental delays in children, behavioural problems of children, and the high number of children present influenced the integration of SuperFIT into daily practice. These barriers appeared especially important because the preschools were situated in low socioeconomic communities.

#### Provision of information

Information provision to the preschool staff was recognised as a limiting factor for the implementation phase. Preschool staff felt they were insufficiently informed about what was expected from them. They also mentioned that they wanted more information about the family component and felt that the preschool and family components were not one integrated programme.

#### Resources

Several factors related to the availability of resources were mentioned for both the implementation and maintenance phase. For the implementation phase, the physical make-up of the room (i.e., available space or arrangement of the room), available time with the children, competing tasks, and high workload were cited. Within the whole organisation, high workload was experienced as a limiting factor for the integration of SuperFIT. With regard to resources in the maintenance phase, specifically the limited availability of funds influenced how SuperFIT could be maintained. Some activities were terminated (e.g., the F&V delivery) and almost all activities needed an alternative form of delivery (e.g., training of the preschool staff). However, managers tried to find solutions to integrate SuperFIT given the limited resources.

### The outer setting

For both the implementation and maintenance phase, collaboration with the primary school was an important influential factor, as most preschools were located within the same building as the primary school. This was experienced as hindering when agreements had to be made about the use of the physical education room or outdoor play area. On the other hand, it was facilitating if the primary school was also actively involved in health promotion. Continuity between the preschool and primary school (e.g., with regard to birthday treats and water drinking policy) was considered important for implementing changes, but also to achieve maintained healthy EBRBs in children.

#### Societal attention

The current societal attention for healthy nutrition and PA in general, but also specific for the ECE setting, was experienced as being supportive. However, both preschool staff and managers felt that most parents did not concern themselves with healthy nutrition or PA in the ECE setting.

#### Rules and regulations

Rules and regulations of the Community Health Authority were considered a limiting factor. Preschool staff felt that rules related to, for example, safety and hygiene limited the possibilities for changes in PA or nutrition.

### Changes in the preschool setting

According to the participants, an increased awareness of the role of preschool staff also led to changes in their behaviour, such as using different types of play materials, more teacher-initiated play, and using different techniques to help preschoolers try new F&V.

#### Nutrition- and physical activity-related practices

This was also reflected in the nutrition- and PA-related practices of the preschool teachers (Table 3). For most practices, a positive change was seen (not statistically tested). Related to PA, the greatest improvements were seen for ‘Modelling’ and ‘Planning time for active play’ at the first follow-up. Other improvements were predominantly moderate and some small. Most changes were still visible at the final follow-up, although they decreased in size. At the first follow-up, a moderate, undesired increase was observed for ‘Not letting the children play out of fear of them getting dirty’, but this did not persist at the final follow-up. Related to nutrition, large improvements were seen for ‘Modelling & Encourage balance and variety’ and ‘Emotion regulation & Food as reward’, all other improvements were mainly moderate. The majority of the improvements persisted over time, some increased (e.g., ‘Involvement & Environment’), while others decreased (e.g., ‘I allow children to help prepare meals’).

**Table 3:** Changes in nutrition- and physical activity-related practices of preschool staff

	Baseline Mean (SD)	T1 Mean (SD)	T2 Mean (SD)	T1 Mean difference	T2 Mean difference
**Physical activity-related practices**					
*Scales (Cronbach’s α)*					
Modelling (.73)	3.92 (0.25)	4.07 (0.35)	3.95 (0.19)	0.15	0.03
Teaching & Autonomy Support (.64)	3.89 (0.26)	3.82 (0.42)	3.85 (0.36)	-0.07	-0.04
Going Outdoors (.52)	4.35 (0.45)	4.22 (0.59)	4.43 (0.36)	-0.13	0.08
*Single items*					
How often do you have outdoor toys for the children (for example skipping ropes, balls)?	3.95 (0.82)	4.31 (0.84)	4.26 (0.56)	0.36	0.31
How often do you keep the children occupied with inactive games?	3.58 (0.43)	3.44 (0.57)	3.37 (0.60)	-0.14	-0.21
How often do you not let children play actively for fear of them getting dirty?	1.09 (0.22)	1.15 (0.31)	1.09 (0.22)	0.06	0.00
How often do you tell children they are not (yet) good enough at sports or active games?	1.05 (0.11)	1.03 (0.10)	1.07 (0.17)	-0.02	0.02
How often do you tell the children that they will get hurt if they play actively?	2.19 (0.78)	2.19 (1.05)	1.94 (0.60)	0.00	-0.25
How often do you discipline children for being too active?	2.82 (0.46)	2.59 (0.66)	2.61 (0.49)	-0.23	-0.21
How often do you reward children for being calm?	2.16 (0.79)	2.10 (0.66)	2.25 (0.73)	-0.06	0.09
How often do you plan time for active play?	4.15 (0.54)	4.47 (0.45)	4.38 (0.33)	0.32	0.23
How often do you keep the children inside despite the weather?	2.29 (0.95)	1.94 (0.95)	1.80 (0.68)	-0.35	-0.49
**Nutrition-related practices**					
*Scales (Cronbach’s α)*					
Modelling and encouraging balance and variety (.84)	4.37 (0.44)	4.76 (0.25)	4.72 (0.39)	0.39	0.35
Involvement and environment (.76)	4.75 (0.24)	4.84 (0.29)	4.90 (0.13)	0.09	0.15
Teaching about nutrition (.69)	3.60 (0.91)	4.07 (0.64)	4.06 (0.82)	0.47	0.46
Pressure to eat (.63)	3.14 (0.66)	3.18 (0.68)	3.04 (0.90)	0.04	-0.10
Emotion regulation and food as reward (.52)	1.25 (0.22)	1.04 (0.08)	1.06 (0.12)	-0.21	-0.19
*Single items*					
How often at meals do you let the children choose the food they want from what is served?	4.18 (0.94)	4.15 (0.97)	4.55 (0.72)	-0.03	0.37
I want to be sure that the children do not eat too many sweets (for example, candy, ice cream, biscuits or pastries).	4.45 (0.70)	3.86 (1.42)	4.38 (0.94)	-0.59	-0.07
I want to be sure that the children do not eat too many high-fat foods (for example, cheese, sausage, cookies).	4.48 (0.73)	4.56 (0.62)	4.10 (1.17)	0.08	-0.38
The children should always eat all the food on their plate.	2.53 (0.85)	2.26 (0.90)	2.02 (0.74)	-0.27	-0.51
I allow the children to help prepare meals (for example, set the table, prepare sandwiches, etc.).	3.62 (1.11)	3.96 (1.00)	3.83 (0.93)	0.34	0.21
I tell the children what to eat and what not to eat without any explanation.	1.54 (0.62)	1.22 (0.46)	1.68 (1.00)	-0.32	0.14

Note: ^a^Items measured on 5-point Likert scale ranging from 1 (Never) to 5 (Always); ^b^Items measured on a 5-point Likert scale ranging from 1 (totally disagree) to 5 (totally agree); *SD* Standard Deviation

#### Social preschool environment

Other changes in the social preschool environment were also observed (Supplementary Table S3). The changes increased over time, which supports preschool staff’s perceptions. Staff started using more play materials, both outdoors and indoors. During indoor play, staff also increasingly initiated activities. However, staff did not initiate outdoor activities. Staff showed more supportive behaviours for PA (e.g., encourage PA). Indoors, staff showed some limiting behaviours for PA (e.g., stimulating children to stay seated) and this did not change over time.

#### Physical preschool environment

Besides the materials that were provided as part of SuperFIT, no major changes were seen in the physical environment (Supplementary Table 4). The availability of portable play materials increased over time in both indoor and outdoor play areas. A decrease was seen in the availability of vegetables at preschools over time, most likely due to termination of the F&V delivery (Supplementary Table S5). Eventually, all preschools switched to only serving water to children.

## Discussion

This process evaluation explored factors influencing implementation and maintenance of the SuperFIT approach and changes the preschool setting. At the start of the implementation phase, predominantly barriers were perceived (e.g., incongruence with current practice, group composition, and the negative attitude of the preschool staff). These barriers are also described for other interventions in the ECE setting [33, 34, 36, 54]. Over time, as staff got more acquainted with the approach, this negative tendency transformed into a more positive view. Although it unclear how much time is exactly needed and this likely differs between interventions and context, sufficient time to implement is thus crucial for successful implementation [55, 56]. Preschool staff participating in SuperFIT needed time to prepare for change. As a result, it may take some time for intervention effects to emerge, and sufficient programme duration and follow-up is essential for detecting these effects [22]. Sequential implementation of intervention components over a longer period of time may assist in implementation and support effectiveness, as staff are able to experience small successes and are not overburdened with intervention activities [56, 57]. The integration of SuperFIT into daily practice was supported by organisational support, increased understanding of the purpose of the approach, and heightened appreciation for the intervention activities from preschool staff. It appeared that over time, SuperFIT increasingly became part of the system in which it was being implemented, which caused contextual factors to become more supportive for integration in practice [30].

An important barrier for implementation and maintenance was the perceived top-down approach of SuperFIT. Although efforts were taken to involve preschool staff (e.g., continued needs assessment and active involvement of the preschool organisation in development), this appeared to be insufficient for staff to feel involved. Involvement of the target group can take several forms, of increasing intensity [58]. From the primary school setting it is known that mutual adaptation processes (i.e., combining top-down and bottom-up approaches) may be essential for successful intervention implementation [59, 60]. To our knowledge, such processes have not yet been described for intervention development in the ECE setting. Although this approach also has barriers (such as time and resources needed), efforts should be taken to increase bottom-up intervention development.

Group composition and characteristics of the children were important barriers throughout the implementation and maintenance phase. Research has shown that chaos at childcare negatively influences the coping responses of childcare staff [61]. This may limit their perceived possibilities to implement SuperFIT elements when groups were perceived as ‘difficult’. Further, research has shown that child characteristics (e.g., child sex and age) influence their EBRBs [62, 63] and also interact with the ECE environment (e.g. child temperament) [64], indicating that different children might need different approaches. Tailoring of interventions to the characteristics of children and groups may support implementation.

Changes in the ECE setting as a result of SuperFIT were predominantly seen in the social environment. Improvements in the nutrition- and PA-related practices of the preschool staff were observed, although these could not be statistically tested due to the small sample size. Staff were also using more play materials and initiating more activities indoors. Effects of other interventions on the social ECE environment have been inconclusive, with some showing changes in the practices of staff while others did not [65–69]. Few changes were seen in the physical environment, except for the SuperFIT materials that were provided. Although intervention studies have been inconclusive, a review showed that changes in the physical environment can evoke large effects on behaviour [70–72]. The importance of the physical environment for EBRBs, also specifically in the ECE setting, has been established [6, 9, 63, 73–76]. More attention for the physical environment (e.g., the outdoor play area) may be supportive to help promote healthy EBRBs in preschoolers. In addition, interaction between the types of environments should be taken into account, since, for example, changes in the physical environment also demand changes in the social environment to have any effect on preschoolers’ EBRBs [40, 77].

An important issue mentioned in this process evaluation was the influence of other settings on the behaviour of preschoolers. Therefore, the role of the preschool setting in preschoolers’ EBRBs was experienced as limited. The home setting (parents) was described as mainly responsible for preschoolers’ EBRBs, in particular related to nutrition. This may be true for preschools in the Netherlands, as they only provide a ‘snack moment’. However, other types of childcare (e.g., full-day childcare) contribute for a large part to the dietary intake of children and attention to healthy nutrition is very important here [78]. Still, the home setting exerts a great influence on child EBRBs for example [79–82]. Furthermore, research has shown that inconsistencies between the ECE and home setting may have negative effects on children’s EBRBs [83]. It remains important to integrate these settings into interventions on preschoolers’ EBRBs to decrease the inconsistencies between the ECE and home settings.

### Implications for implementation

Our study identified some implications for implementation research. First, it appeared important to take sufficient time before starting the implementation to get all stakeholders involved on the same page. Providing information on the programme, but also making sure the required preconditions are met (e.g., reimbursement of invested time) is essential to take into account in this preparation phase. Second, intervention implementation should not be considered a temporary effort. To increase integration within practice and thus intervention success long-term commitment is needed. This may be facilitated through cooperation with practice partners who are able to have this long-term commitment. This may not always be possible within research institutions. Third, a participatory approach in development and implementation can increase the acceptability of the changes that the intervention aims to achieve. Last, for the success of implementation it is important to be open-minded to adaptations to the programme to support the fit to the local setting [84].

### Strengths and limitations of the study

The mixed-methods design of this study made data triangulation possible, by integrating the quantitative and qualitative data in the interpretation phase. Both quantitative and qualitative data were available and ensured an elaborate understanding of processes that influenced implementation and maintenance. This process evaluation also goes beyond studying the ‘quantitative’ concepts that are more traditionally used to describe implementation. The theoretical framework that was adopted supported this more elaborate study of implementation and maintenance, including context. Furthermore, various stakeholders were included in the study, which made it possible to study the implementation and maintenance of SuperFIT from different perspectives. Research methods were flexible, which enabled the researcher to, for example, add interview rounds when it became evident that this would increase the understanding of implementation and maintenance.

This study describes the factors that influenced implementation and maintenance of SuperFIT, which was implemented in a specific region in the Netherlands. Results of this study may not be generalisable to other intervention programmes or regions. However, the lessons learned from this study may be valuable for all intervention developers and implementers. Furthermore, the importance of a contextual approach to intervention development and implementation is highlighted, which takes into account the specific contextual factors that may be of influence in a particular region or for a specific organisation [30, 31]. The quantitative analyses were performed on a preschool level, which resulted in a small sample size (*N*=12). Therefore, no statistical testing was possible. For the observational data, nine preschool (10 groups) were included. Observations were ended after saturation had occurred to support feasibility of the data collection. Selection bias may have occurred in the recruitment of the preschool teachers for the interviews. These were performed on a voluntary basis and this may have resulted in the participation of preschool teachers with a more positive view on SuperFIT. However, the quantitative process evaluation was performed among all preschool teachers participating in SuperFIT.

## Conclusion

Several factors influenced the implementation and maintenance of the SuperFIT approach in preschools. Over time, some of these factors changed from barriers to facilitators, indicating the importance of allowing sufficient time for implementation and follow-up to be able to initiate and detect change. Changes mainly occurred in the social environment. An important perceived change was improved awareness of the preschool staff of their influence on preschoolers’ EBRBs. This may be a prerequisite for behavioural changes to occur and indicates the importance of involvement of preschool staff in the early phases of intervention development. Bottom-up or mutual adaptation approaches may support this, although active involvement of preschool staff is required, which may be regarded as a barrier for such approaches.

## Supplementary Information

**Additional file 1.**

**Additional file 2.**

**Additional file 3.**

**Additional file 4.**

**Additional file 5.**

**Additional file 6.**

**Additional file 7.**

## Data Availability

The datasets used and/or analysed during the current study are available from the corresponding author on reasonable request.

## Abbreviations

CFAPQ: Child-care Food and Activity Practices Questionnaire
CFIR: Consolidated Framework for Implementation Research
EBRBs: Energy Balance-Related Behaviours
ECE: Early Care and Education
EPAO: Environment and Policy Assessment and Observation
F&V: Fruit and Vegetables
MIDI: Measurement Instrument for Determinants of Innovations
PA: Physical Activity
