# Supplementary material for: Changing the preschool setting to promote healthy energy balance-related behaviours of preschoolers: a qualitative and quantitative process evaluation of the SuperFIT approach
Source: Implement Sci. 2021 Dec 4;16:101. doi: 10.1186/s13012-021-01161-9 (PMC8642927; doi:10.1186/s13012-021-01161-9)
Supplement: Supplementary file 2 — Additional file 2. [file 13012_2021_1161_MOESM2_ESM.docx]

Supplementary Table S1. Use of SuperFIT materials during implementation and maintenance within the intervention preschools.

| Variable | 1^st^ observation (implementation) | 2^nd^ observation (implementation) | 3^rd^ observation (maintenance) |
| --- | --- | --- | --- |
| *Physical activity* | | | |
| Number of materials visible^a^ (min/max, mean± SD) | 0/3, 1.20± 1.23 | 0/5, 1.80± 1.69 | 0/8, 2.00± 2.40 |
| Number of locations with PA materials used | 7 | 8 | 9 |
| Number of materials used by staff^a^ (min/max, mean± SD) | 0/3, 1.10± 1.10 | 0/4, 0.90± 1.29 | 0/4, 1.20± 1.62 |
| Number of materials used by children^a^ (min/max, mean± SD) | 0/3, 1.20± 1.14 | 0/4, 1.40± 1.17 | 0/3, 1.20± 1.03 |
| PA-related cards used by staff (yes/no) | 0/10 | 1/9 | 4/6 |
| *Nutrition* | | | |
| Number of locations with nutrition materials used | 8 | 7 | 7 |
| Number of materials visible^a^ (min/max, mean± SD) | 0/4, 1.30± 1.16 | 0/2, 0.60± 0.70 | 0/4, 1.60± 1.43 |
| Number of materials used by staff^a^ (min/max, mean± SD) | 0/1, 0.40± 0.52 | 0/1, 0.30± 0.48 | 0/1, 0.30± 0.48 |
| Number of materials used by children^a^ (min/max, mean± SD) | 0/2, 0.80± 0.92 | 0/3, 1.10± 0.99 | 0/3, 1.00± 0.94 |
| Nutrition-related cards used by staff (yes/no) | 0/10 | 1/9 | 1/9 |

Note: 1^st^, 2^nd^ and 3^rd^ observations were performed in September/October 2017, April 2018, and September 2018 respectively; ^a^ visibility, use by staff and use by children are based on all preschools, zero reflects no visibility or use of the materials; max. = maximum, min = minimum, SD = standard deviation.
