# Supplementary material for: Changing the preschool setting to promote healthy energy balance-related behaviours of preschoolers: a qualitative and quantitative process evaluation of the SuperFIT approach
Source: Implement Sci. 2021 Dec 4;16:101. doi: 10.1186/s13012-021-01161-9 (PMC8642927; doi:10.1186/s13012-021-01161-9)
Supplement: Supplementary file 3 — Additional file 3. [file 13012_2021_1161_MOESM3_ESM.docx]

Supplementary Table S2. Quantitative process evaluation of SuperFIT from the preschool teachers.

| **Process questionnaire regarding implementation** | **Mean** | **SD** |
| --- | --- | --- |
| *The SuperFIT intervention programme* | | |
| What do you think of SuperFIT in general?^a^ | 3.96 | 0.73 |
| Did you find SuperFIT interesting?^b^ | 3.84 | 0.80 |
| Did you find SuperFIT educational?^c^ | 3.76 | 0.83 |
| Did you find SuperFIT clear?^d^ | 4.08 | 0.86 |
| Are you satisfied with the communication about SuperFIT?^e^ | 3.72 | 0.94 |
| What did you think about the amount of time SuperFIT cost you?^f^ | 3.44 | 0.87 |
| Did SuperFIT meet your expectations?^g^ | 3.44 | 0.96 |
| Do you think that SuperFIT helped you to better address the nutrition and physical activity of preschoolers?^h^ | 3.56 | 0.92 |
| Do you think that SuperFIT has led to a healthier lifestyle of you personally?^h^ | 2.28 | 1.17 |
| Do you think that the preschoolers are more physically active due to SuperFIT?^h^ | 3.60 | 1.12 |
| Do you think that the preschoolers eat more healthily due to SuperFIT?^h^ | 3.28 | 1.10 |
| Do you feel you use a more positive way in addressing the preschoolers?^h^ | 3.04 | 1.10 |
|  | | |
| *Training off-the-job and coaching on-the-job* | | |
| Did you find the materials used during the training off-the-job interesting?^b^ | 3.72 | 0.79 |
| Did you find the materials used during the training off-the-job clear?^d^ | 4.08 | 0.70 |
| Did you find the trainer on nutrition qualified?^i^ | 4.20 | 0.83 |
| Did you find the trainer on physical activity qualified?^i^ | 4.28 | 0.79 |
| Did you find the trainer on positive parenting qualified?^i^ | 4.16 | 0.85 |
| What did you think about the group-size during the training off-the-job?^j^ | 3.28 | 0.46 |
| Did you find the coaching on-the-job useful?^c^ | 3.64 | 0.86 |
| Did you find the coach on physical activity qualified?^i^ | 4.20 | 0.87 |
| Did you find the coach on nutrition qualified?^i^ | 4.18 | 0.73 |
| Did you find the coaching on-the-job fit complemented the training off-the-job?^h^ | 4.48 | 0.28 |
|  | | |
| *Fruit and vegetables delivery* | | |
| What do you think about the fruit and vegetables delivery in general?^a^ | 4.38 | 0.80 |
| What did you think about the variety of the fruit and vegetables delivery?^k^ | 4.62 | 0.54 |
| What did you think about the amount of the fruit and vegetables delivered?^l^ | 2.98 | 0.55 |
| Do you find preparing the fruit and vegetables difficult?^m^ | 3.56 | 0.85 |
| How much extra time does it cost you to prepare the fruit and vegetables?^n^ | 3.46 | 0.71 |
| Did you find it difficult to let the preschoolers eat the new fruit and vegetables?^m^ | 3.16 | 0.88 |
| How often did the preschoolers eat the new fruit and vegetables?^o^ | 3.54 | 0.71 |
|  | | |
| *General material box and nutrition and physical activity cards* | | |
| Do you find the materials of the general material box useful?^c^ | 4.28 | 0.89 |
| Do you find the materials of the general material box easy to use?^m^ | 4.08 | 0.64 |
| Do you find the materials appropriate for the preschool?^p^ | 4.16 | 0.69 |
| How often do you use the materials of the general material box?^o^ | 3.72 | 0.54 |
| What do you think of the nutrition and physical activity cards?^a^ | 3.48 | 0.82 |
| Do you find the nutrition and physical activity cards useful?^c^ | 3.64 | 0.81 |
| Do you find the nutrition and physical activity cards easy to use?^m^ | 3.52 | 0.71 |
| Do you find the nutrition and physical activity cards appropriate for preschools?^p^ | 3.24 | 0.83 |
| How often do you use the nutrition and physical activity cards?^o^ | 3.20 | 0.65 |

Anwering scales: ^a^really bad (1) to really good (5), ^b^very uninteresting (1) to very interesting (5), ^c^very useless (1) to very useful (5), ^d^very unclear (1) to very clear (5), ^e^very dissatisfied (1) to very satisfied (5), ^f^worse than expected (1) to better than expected (5), ^g^none of the expectations (1) to almost all of the expectations (5), ^h^certainly not (1) to certainly (5), ^i^very inappropriate (1) to very appropriate (5), ^j^too small (1) to too large (5), ^k^not at all varied (1) to very varied (5), ^l^too little (1) to too much (5), ^m^very difficult (1) to very easy (5), ^n^no extra time (1) to a lot of extra time (5), ^o^never (1) to always (5), ^p^very inappropriate (1) to very appropriate (5). SD = standard deviation

| **Process questionnaire regarding implementation/maintenance** | **Mean** | **SD** |
| --- | --- | --- |
| SuperFIT is too complicated for me to implement.^a^ | 1.68 | 0.69 |
| I think the principles of SuperFIT are right.^a^ | 3.92 | 0.91 |
| SuperFIT fits well with my daily practice.^a^ | 3.88 | 0.97 |
| I think SuperFIT is appropriate for preschoolers.^a^ | 4.00 | 0.91 |
| I have enough knowledge to implement SuperFIT.^a^ | 4.24 | 0.83 |
| I know enough about the content of SuperFIT.^a^ | 4.24 | 0.83 |
| I think it is part of my job to implement SuperFIT.^a^ | 3.88 | 0.88 |
| Implementing SuperFIT has disadvantages for me personally.^a^ | 2.32 | 0.95 |
| Implementing SuperFIT has advantages for me personally.^a^ | 3.68 | 0.95 |
| I think preschoolers are healthier due to SuperFIT.^a^ | 3.48 | 1.05 |
| I can count on the help of my colleagues in implementing SuperFIT.^a^ | 4.20 | 0.87 |
| I can count on the help of my manager in implementing SuperFIT.^a^ | 3.84 | 0.90 |
| How many of your colleagues for which SuperFIT was intended, also implement SuperFIT according to you?^b^ | 3.44 | 1.00 |
| To what extent do your colleagues expect you to implement SuperFIT?^c^ | 3.84 | 0.69 |
| To what extent does your manager expect you to implement SuperFIT?^c^ | 4.28 | 0.54 |
| To what extent does the management of your organisation expect you to implement SuperFIT?^c^ | 4.24 | 0.52 |
| To what extent do parents of the preschoolers expect you to implement SuperFIT?^c^ | 3.04 | 0.89 |
| I feel like I can implement the principles of SuperFIT in my daily work.^c^ | 4.20 | 0.58 |
| My organisation has taken measures to ensure implementation of SuperFIT by new employees.^a^ | 2.88 | 0.60 |
| My organisation has sufficient staff to implement SuperFIT.^a^ | 3.52 | 0.59 |
| There are sufficient financial resources to implement SuperFIT.^a^ | 3.00 | 0.87 |
| My organisation ensures that I have enough time available to implement SuperFIT.^a^ | 3.08 | 0.81 |
| My organisation ensures that I have sufficient materials and services available to implement SuperFIT.^a^ | 3.24 | 1.01 |
| There is regular feedback on the implementation of SuperFIT with my manager.^a^ | 3.00 | 1.00 |
| I have access to information on the implementation of SuperFIT within my organisation.^a^ | 3.40 | 0.71 |
|  | **Yes%** | **No%** |
| Are there formal agreements (policies or work instructions) made by the management of your organisation? | 36% | 64% |
| Were there any other changes within your organisation during the implementation of SuperFIT (reconstitution, mergers, cuts, or turnover)? | 20% | 80% |
| Were there, besides SuperFIT, any other innovations during the implementation of SuperFIT? | 16% | 84% |

Answering scales: ^a^totally disagree (1) to totally agree (5), ^b^none of my colleagues (1) to all of my colleagues (5), ^c^most certainly not (1) to certainly (5). SD = standard deviation
