# Supplementary material for: Changing the preschool setting to promote healthy energy balance-related behaviours of preschoolers: a qualitative and quantitative process evaluation of the SuperFIT approach
Source: Implement Sci. 2021 Dec 4;16:101. doi: 10.1186/s13012-021-01161-9 (PMC8642927; doi:10.1186/s13012-021-01161-9)
Supplement: Supplementary file 4 — Additional file 4. [file 13012_2021_1161_MOESM4_ESM.docx]

Supplementary Table S3. The physical activity- and nutrition-related social environment at the intervention preschools of SuperFIT (N=10).

|  | 1^st^ observation (implementation) | 2^nd^ observation (implementation) | 3^rd^ observation  (Maintenance) |
| --- | --- | --- | --- |
| *Physical Activity* | | | |
| Outdoor play (Yes/No)^a^ | 7/3 | 7/3 | 7/3 |
| Teacher-initiated play outdoors (Yes/No)^a^ | 4/6 | 0/10 | 2/8 |
| Outdoor play materials (Yes/No)^a^ | 7/3 | 7/3 | 7/3 |
| Number of used outdoor play materials (min/max, mean± SD) | 0/5, 1.70± 1.77 | 0/6, 2.10± 1.97 | 0/6, 2.70± 2.06 |
| Outdoor supportive staff behaviour (min/max, mean± SD) | 0/4, 2.43± 1.99 | 0/3, 0.57± 1.13 | 1/6, 2.71± 1.70 |
| Outdoor limiting staff behaviour (Yes/No)^b^ | 0/7 | 0/7 | 0/7 |
| Supportive social environment outdoors (min/max, mean± SD)^c^ | 1/5, 3.43± 1.99 | 1/4, 1.57± 1.13 | 2/10, 4.29± 2.75 |
| Indoor play (Yes/No)^a^ | 10/0 | 10/0 | 10/0 |
| Teacher-initiated play indoors (Yes/No)^a^ | 8/2 | 7/3 | 9/1 |
| Number of used indoor play materials (min/max, mean± SD) | 0/5, 2.10± 1.52 | 1/6, 3.50± 1.58 | 0/7, 2.80± 2.39 |
| Indoor supportive staff behaviour (min/max, mean± SD) | 2/6, 3.90± 1.37 | 1/9, 4.10± 2.38 | 0/9, 5.20± 2.39 |
| Indoor limiting staff behaviour (min/max, mean± SD) | 0/5, 3.20± 1.32 | 2/5, 2.80± 1.03 | 1/5, 3.30± 1.16 |
| Supportive social environment indoors (min/max, mean± SD)^c^ | 2/8, 5.20± 2.35 | 1/13, 5.30± 3.43 | 2/12, 6.80± 2.78 |
| *Nutrition* | | | |
| Supportive staff behaviour (min/max, mean± SD) | 1/6, 4.30± 1.64 | 2/6, 4.50± 1.65 | 2/6, 4.10± 1.37 |
| Non-supportive staff behaviour (min/max, mean± SD) | 0/1, 0.20± 0.42 | 0/0 | 0/1, 0.40± 0.42 |
| Supportive social environment^c^ (min/max, mean± SD) | 5/10, 8.10± 1.73 | 6/10, 8.50± 1.65 | 5/10, 7.70± 1.57 |
| Strategies used to promote tasting (min/max, mean± SD) | 1/5, 2.75± 1.58 | 1/4, 2.13± 1.36 | 1/3, 2.00± 0.63 |

Note: 1^st^, 2^nd^ and 3^rd^ observations were performed in September/October 2017, April 2018, and September 2018 respectively; ^a^Scores reflect activities at preschool level; ^b^Scores are based on one question; ^c^Scores are based on the combination of supporting and limiting staff behaviour, where limiting behaviours were recoded in order to calculate an overall positive score; max. = maximum, min = minimum, SD = standard deviation.
