# Supplementary material for: Changing the preschool setting to promote healthy energy balance-related behaviours of preschoolers: a qualitative and quantitative process evaluation of the SuperFIT approach
Source: Implement Sci. 2021 Dec 4;16:101. doi: 10.1186/s13012-021-01161-9 (PMC8642927; doi:10.1186/s13012-021-01161-9)
Supplement: Supplementary file 5 — Additional file 5. [file 13012_2021_1161_MOESM5_ESM.docx]

Supplementary Table S4. The physical activity-related physical environment at the intervention preschools of SuperFIT (N=12).

|  | 1^st^ measurement | 2^nd^ measurement | 3^rd^ measurement |
| --- | --- | --- | --- |
| Fixed play equipment available outdoors (mean types± SD) | 4.00± 2.71 | 4.33± 2.61 | 4.50± 2.75 |
| Portable play equipment available outdoors (mean types± SD) | 5.90± 1.73 | 4.75± 2.30 | 6.25± 2.30 |
| Fixed play equipment available indoors (mean types± SD) | 3.90± 1.66 | 3.00± 1.95 | 2.67± 1.37 |
| Portable play equipment available indoors (mean types± SD) | 8.60± 1.58 | 8.00± 3.22 | 8.83ׅ± 3.46 |
| Screens and electrical devices (TV, DVD, smartboard) available (mean± SD) | 1.70± 1.16 | 1.42± 1.09 | 1.08± 0.90 |

Note: SD= standard deviation
