# Supplementary material for: Changing the preschool setting to promote healthy energy balance-related behaviours of preschoolers: a qualitative and quantitative process evaluation of the SuperFIT approach
Source: Implement Sci. 2021 Dec 4;16:101. doi: 10.1186/s13012-021-01161-9 (PMC8642927; doi:10.1186/s13012-021-01161-9)
Supplement: Supplementary file 6 — Additional file 6. [file 13012_2021_1161_MOESM6_ESM.docx]

Supplementary Table S5. The nutrition-related physical environment during the observations at the intervention preschools of SuperFIT (N=10).

|  | 1^st^ observation (implementation) | 2^nd^ observation (implementation) | 3^rd^ observation (maintenance) |
| --- | --- | --- | --- |
| Availability of food at number of preschools  Fruit  Vegetables | 10  6 | 10  4 | 10  3 |
| Different types of fruit (min/max, mean± SD) | 3/7, 4.70± 1.16 | 4/6, 5.20± 0.79 | 4/6, 4.40± 0.84 |
| Different types of vegetables (min/max, mean± SD) | 1/3, 1.50± 0.84 | 1/3, 1.80± 1.10 | 1/2, 1.67± 0.58 |
| Availability of drinks at number of preschools  Water  Lemonade | 9  1 | 9  1 | 10  0 |

Note: 1^st^, 2^nd^ and 3^rd^ observations were performed in September/October 2017, April 2018, and September 2018 respectively; max. = maximum, min = minimum, SD = standard deviation.
