# Supplementary material for: Changing the preschool setting to promote healthy energy balance-related behaviours of preschoolers: a qualitative and quantitative process evaluation of the SuperFIT approach
Source: Implement Sci. 2021 Dec 4;16:101. doi: 10.1186/s13012-021-01161-9 (PMC8642927; doi:10.1186/s13012-021-01161-9)
Supplement: Supplementary file 7 — Additional file 7. [file 13012_2021_1161_MOESM7_ESM.docx]

**Supplementary material – Topic Lists**

***Interviews preschool teachers – June/July 2017***

- General
  - What are your experiences with SuperFIT up until now?
    - Are you able to integrate in daily practice?
    - How do you feel about communication?
  - What do you find positive about SuperFIT?
  - What could be improved?
- Fruit and vegetables delivery
  - What are your experiences with the fruit and vegetables delivery?
  - What do you find positive about the fruit and vegetables delivery?
  - What could be improved?
- Off-the-Job training
  - Where you present at the off-the-job training? Why so/not?
  - What were your experiences with the off-the-job training?
    - Content
    - Implementers
  - What was positive about the training?
  - What could be improved?
- On-the-job coaching
  - Did you receive on-the-job coaching? Why not?
  - What were your experiences with the on-the-job coaching?
    - Content
    - Implementer
  - What was positive about the coaching?
  - What could be improved?
- Additional materials and cards
  - How do you appreciate the additional materials and cards?
    - Did you already use them? Why so/not?
  - What do you find positive about the additional materials and cards?
    - What materials/cards do you use often?
    - What materials/cards do the children like?
  - What is less positive about the additional materials and cards?
    - Are there materials that you haven’t used yet?
    - Are you able to store the materials?
- Specific needs for materials
  - Are there any materials lacking (after using the additional materials)?
  - Do you have any need for (play) materials?
    - What can we do to fulfill that need?
  - Do you have any other needs for changes at your preschool?
    - Other additional materials
    - Changes in the physical environment
    - Changes in other types of environments (e.g., policy)

***Interviews preschool teachers January/February 2018***

I would like to start with looking back at the activities of SuperFIT that took place after summer holidays.

- Fruit and vegetables delivery
  - What were your experiences with the fruit and vegetables delivery the past period?
  - Does something need to change in the fruit and vegetables delivery?
- Off-the-job training
  - Were you present at the off-the-job training?
  - What do you think of the two off-the-job trainings after the summer?
- On-the-job coaching
  - Did you receive tow coaching on-the-job? On which subjects?
  - What do you think of the coaching on-the-job after the summer?
- Additional materials and cards
  - Do you use the additional materials and cards in daily practice?
    - Why so/not?
    - When do you use them or not use them?
  - What would help you to make more use of the additional materials and cards?

Now I would like to look back at SuperFIT in general

- Strengths
  - What did you find strengths of SuperFIT?
    - Were there any other positive things about SuperFIT?
    - Can you name something of SuperFIT that you use in daily practice?
    - Are there things that you left out of daily practice, in order to integrate SuperFIT activities?
  - What do the children think about SuperFIT (additional materials/activities/fruit and vegetables)?
  - What parts of SuperFIT were most helpful to work on the goals of SuperFIT (more physical activity, less sedentary behavior, drinking water, and healthier dietary intake)?
- Weaknesses
  - Can you name weaknesses of SuperFIT?
    - What did you find less positive about SuperFIT?
  - Which SuperFIT activities did you consider less helpful?
- Opportunities
  - Do you see opportunities for SuperFIT within your organization?
  - Do you see opportunities in your daily practice that can be used to work on the goals of SuperFIT (more physical activity, less sedentary behavior, drinking water, and healthier dietary intake)?
- Threats
  - Can you think of threats for integration of SuperFIT, for example, in your organization or in daily practice?
- SuperFIT in daily practice
  - Do you experience changes in your daily practice due to SuperFIT (e.g., your own behavior, in the children, in colleagues)?
    - Did your own attitude on nutrition and physical activity change?
    - To what extent is SuperFIT integrated in daily practice?
      - Does it take extra time to integrate SuperFIT?
  - Are there things unchanged in daily practice, that were part of SuperFIT?
    - Were the training off-the-job/coaching on-the-job sufficient for change? If not, what would you need?
    - Did you quit with SuperFIT activities that you took up? Why?
  - Do you experience changes in the parents regarding nutrition and physical activity?
    - Did you try to involve parents more on these subjects?
    - What could be done to involve parents more?

Finally, I would like to talk about some general experiences with SuperFIT.

- Communication
  - How do you feel about the communication regarding SuperFIT?
    - Did you get information from whom you expected to receive information?
- Involvement of preschool teacher in development of SuperFIT
  - How do you feel about how preschool teachers were involved in the development of SuperFIT?
    - What could have been done differently? How?
  - Do you feel like the SuperFIT activities were adaptable to your local situation? Why so/not?
  - Would you be willing to participate in a workgroup or so on SuperFIT?
- Support from the organization
  - Do you feel that SuperFIT was supported from the management of your organization? Why so/not?
  - Do you feel that you as preschool teacher were supported by your organization to integrate SuperFIT? Why so/not?
    - What do you need to feel supported?
  - Do you feel things changed within your organization to support you in the integration of SuperFIT in daily practice?
    - What would need to change?
- Maintenance/dissemination
  - What do you think is important to maintain of SuperFIT within your organization?
  - If SuperFIT were to be disseminated to other preschools, what would be your recommendations?

***Interviews Preschool Teachers October/November 2018***

**Maintenance SuperFIT**

I would like to talk to you about what SuperFIT looks like in your daily practice.

- What does SuperFIT currently look like at your preschool?
  - What do you still use or not? Why?
  - Did you receive any additional materials?
    - If yes, how do you feel about them?
    - If no, why not?
  - What do you feel is the essence of SuperFIT?
- Which factors still influence healthy nutrition and physical activity at your preschool?
  - How do you handle them?
  - Were there any changes herein during the last year?
- What is, in your opinion, needed to maintain SuperFIT?
- Is there any attention within your organization towards maintenance of SuperFIT? If yes, how?
  - How does your organization handle new colleagues regarding SuperFIT?
  - Do you have specific needs towards your organization to be able to maintain SuperFIT at your preschool?

**Preschool teachers’ part in healthy nutrition and physical activity**

I would like to talk more in-depth about how you think about healthy nutrition and physical activity in preschoolers in general.

- How do you see your part in healthy nutrition and physical activity in preschoolers?
  - Is there any difference between nutrition and physical activity?
  - Are there influences in how you can play this part?
  - How does it relate to other tasks you have to fulfil as a professional?
  - Does SuperFIT help you in fulfilling that part?
- Which other people play a part in healthy nutrition and physical activity in preschoolers?
  - Within your organization? Outside your organization?
  - Do they influence your part? If yes, how?
  - Was there sufficient attention within SuperFIT on this? What would you have liked to see differently?

**Cognitive variables**

- Attitude
  - How do you feel about healthy nutrition and physical activity in preschools?
    - Why do you think it is/is not important?
    - Did SuperFIT change your views? If yes, how? If not, why not?
      - Are you more aware on healthy nutrition and physical activity within your work?
- Subjective norm
  - Are there people within your (work)environment that expect you to be involved with healthy nutrition and physical activity in preschoolers?
    - Do you find it important to live up to that expectation? Why so/not?
  - Do you feel people within your organization are involved in healthy nutrition and physical activity of preschoolers?
    - What was the part of SuperFIT in this?
- Perceived behavioral control
  - Do you feel capable to integrate healthy nutrition and physical activity within your daily practice?
  - Do you feel SuperFIT can help in changing the energy balance-related behaviors of preschoolers? Why so/not?
  - Can preschools, in your opinion, influence the energy balance-related behaviors of preschoolers?
- Intention
  - Do you intend to stay involved in healthy nutrition and physical activity in your work?
    - Do you think it belongs to your tasks? Why so/not?
      - How did SuperFIT influence this?

***Interviews Management October/November 2018***

**Maintenance SuperFIT**

- What do you think is SuperFIT?
  - What do you think is the essence of SuperFIT?
  - What do you think are strengths of SuperFIT?
  - What do you think are weaknesses?
- Do you think that you have enough knowledge on the content of SuperFIT?
- What do see as your part within SuperFIT?
- How do you look back at the implementation of SuperFIT?
  - What changes do you perceive in the preschools? And within your organization?
  - Why do you think some activities were successful or less successful?
  - Were there other things happening within your organization?
- What is being done to maintain SuperFIT within the organization?
  - New preschool teachers?
- What do you think is needed to maintain SuperFIT within your organization?
  - Which factor influence this?
  - Do you see opportunities or threats?

**Management’s part in healthy nutrition and physical activity**

- Which factors influence healthy nutrition and physical activity in preschools?
  - Do you perceive any changes?
  - What part did SuperFIT play herein?
- How do you see your part in healthy nutrition and physical activity in preschoolers?
  - Is there any difference between nutrition and physical activity?
  - Are there influences in how you can play this part?
  - How does it relate to other tasks you have to fulfil as a professional?
  - Does SuperFIT help you in fulfilling that part?
- Which other people play a part in healthy nutrition and physical activity in preschoolers?
  - Within your organization? Outside your organization?
  - Do they influence your part? If yes, how?
  - Was there sufficient attention within SuperFIT on this? What would you have liked to see differently?

**Cognitive variables**

- Attitude
  - How do you feel about healthy nutrition and physical activity in preschools?
    - Why do you think it is/is not important?
    - Did SuperFIT change your views? If yes, how? If not, why not?
      - Are you more aware on healthy nutrition and physical activity within your work?
- Subjective norm
  - Are there people within your (work)environment that expect you to be involved with healthy nutrition and physical activity in preschoolers?
    - Do you find it important to live up to that expectation? Why so/not?
  - Do you feel people within your organization are involved in healthy nutrition and physical activity of preschoolers?
    - What was the part of SuperFIT in this?
- Perceived behavioral control
  - Do you feel capable to integrate healthy nutrition and physical activity within your daily practice?
  - Do you feel SuperFIT can help in changing the energy balance-related behaviors of preschoolers? Why so/not?
  - Can preschools, in your opinion, influence the energy balance-related behaviors of preschoolers?
- Intention
  - Do you intend to stay involved in healthy nutrition and physical activity in your work?
    - Do you think it belongs to your tasks? Why so/not?
      - How did SuperFIT influence this?

***Interviews Implementers October/November 2018***

**Maintenance SuperFIT**

- What do you think is SuperFIT?
  - What do you think is the essence of SuperFIT?
  - What do you think are strengths of SuperFIT?
  - What do you think are weaknesses?
- How do you look back at the implementation of SuperFIT?
  - What changes do you perceive in the preschools? And within your organization?
  - Why do you think some activities were successful or less successful?
  - Were there other things happening within your organization?
- What is being done to maintain SuperFIT within the organization?
  - New employees?
- What do you think is needed to maintain SuperFIT within your organization?
  - Which factor influence this?
  - Do you see opportunities or threats?

**Management’s part in healthy nutrition and physical activity**

- Which factors influence healthy nutrition and physical activity in preschools?
  - Do you perceive any changes?
  - What part did SuperFIT play herein?
- How do you see your part in healthy nutrition and physical activity in preschoolers?
  - Is there any difference between nutrition and physical activity?
  - Are there influences in how you can play this part?
  - How does it relate to other tasks you have to fulfil as a professional?
  - Does SuperFIT help you in fulfilling that part?
- Which other people play a part in healthy nutrition and physical activity in preschoolers?
  - Within your organization? Outside your organization?
  - Do they influence your part? If yes, how?
  - Was there sufficient attention within SuperFIT on this? What would you have liked to see differently?

**Cognitive variables**

- Attitude
  - How do you feel about healthy nutrition and physical activity in preschools?
    - Why do you think it is/is not important?
    - Did SuperFIT change your views? If yes, how? If not, why not?
      - Are you more aware on healthy nutrition and physical activity within your work?
- Subjective norm
  - Are there people within your (work)environment that expect you to be involved with healthy nutrition and physical activity in preschoolers?
    - Do you find it important to live up to that expectation? Why so/not?
  - Do you feel people within your organization are involved in healthy nutrition and physical activity of preschoolers?
    - What was the part of SuperFIT in this?
- Perceived behavioral control
  - Do you feel capable to integrate healthy nutrition and physical activity within your daily practice?
  - Do you feel SuperFIT can help in changing the energy balance-related behaviors of preschoolers? Why so/not?
  - Can preschools, in your opinion, influence the energy balance-related behaviors of preschoolers?
- Intention
  - Do you intend to stay involved in healthy nutrition and physical activity in your work?
    - Do you think it belongs to your tasks? Why so/not?
      - How did SuperFIT influence this?
